# Leveraging Deep Neural Networks for Estimating Vickers Hardness from Nanoindentation Hardness

**DOI:** 10.3390/ma17010148

**Published:** 2023-12-27

**Authors:** Junbo Niu, Bin Miao, Jiaxu Guo, Zhifeng Ding, Yin He, Zhiyu Chi, Feilong Wang, Xinxin Ma

**Affiliations:** 1 School of Material Science & Engineering, Harbin Institute of Technology, Harbin 150001, China; 18b309004@stu.hit.edu.cn (B.M.); 20b909021@stu.hit.edu.cn (J.G.); 21b909019@stu.hit.edu.cn (Z.D.); 23b909030@stu.hit.edu.cn (Y.H.); 22s009074@stu.hit.edu.cn (Z.C.); 22s109197@stu.hit.edu.cn (F.W.); 2 State Key Laboratory of Advanced Welding and Joining, Harbin Institute of Technology, Harbin 150001, China

**Keywords:** deep neural network, hardness, nanoindentation, nitriding

## Abstract

This research presents a comprehensive analysis of deep neural network models (DNNs) for the precise prediction of Vickers hardness (HV) in nitrided and carburized M50NiL steel samples, with hardness values spanning from 400 to 1000 HV. By conducting rigorous experimentation and obtaining corresponding nanoindentation data, we evaluated the performance of four distinct neural network architectures: Multilayer Perceptron (MLP), Convolutional Neural Network (CNN), Long Short-Term Memory network (LSTM), and Transformer. Our findings reveal that MLP and LSTM models excel in predictive accuracy and efficiency, with MLP showing exceptional iteration efficiency and predictive precision. The study validates models for broad application in various steel types and confirms nanoindentation as an effective direct measure for HV hardness in thin films and gradient-variable regions. This work contributes a validated and versatile approach to the hardness assessment of thin-film materials and those with intricate microstructures, enhancing material characterization and potential application in advanced material engineering.

## 1. Introduction

The hardness of a material serves as a crucial index, encapsulating the material’s capacity to resist deformation or fracturing, bearing immense implications for disciplines such as materials science, engineering, and manufacturing [1,2,3,4,5,6,7,8,9,10,11,12]. Methods to measure hardness, embracing Vickers hardness (HV) [13,14,15,16], Brinell hardness (HB) [17,18,19,20], and Rockwell hardness (HR) [21,22,23,24], have garnered wide acceptance, contributing a valuable perspective in the comprehension of materials’ mechanical behaviors. Within the ambit of materials science, the process of hardness conversion is of paramount importance [25,26,27]. It unearths not only the deformation-resistance properties of an identical material across varied hardness scales but also introduces the opportunity to evaluate additional mechanical properties under non-destructive circumstances, thus bypassing more invasive testing approaches. Moreover, hardness conversion extends essential references for the selection and design of materials across a spectrum of application requirements. Crucially, whether the materials’ scales are macroscopic, microscopic, or nanoscale, hardness conversion enables the performance comparison across these scales, thus advancing a deeper interpretation and evaluation of material performance. Researchers [27] have developed theoretical equations to transition between Brinell hardness (HB), Rockwell hardness (HR), and Vickers hardness (HV). These equations are based on the power-law stress-strain relationship and the equivalent energy principle. Furthermore, the pre-existing relationship between tensile strength (*σ_b_*) and Hollomon parameters (K, N) was integrated, allowing the derivation of the theoretical conversion between hardness (HB/HR/HV) and tensile strength (*σ_b_*). These equations have undergone rigorous validation using an extensive dataset from ASTM and ISO standards, demonstrating a high degree of agreement.

However, these conventional hardness-characterization techniques may incur destructive consequences and require substantial time. Moreover, the precise hardness quantification of thin films and coatings poses significant challenges, which restrict the application of these measurement methodologies in such scenarios. With the advent of nanoindentation technology, it is now feasible to gauge hardness at the nanoscale. This innovative method presents a more efficient and less destructive substitute for thin films and coatings [28,29,30,31]. Determining the relationship between the comparatively microscopic nanohardness and macrohardness proves to be exceptionally crucial. However, the connection between Vickers hardness and nanoindentation hardness is far from linear, with multiple factors such as the material properties and microstructures exerting substantial influence. Certain researchers [26] observed in their investigations of Vickers hardness and nanoindentation hardness of unalloyed titanium (Ti), nickel (Ni), tungsten (W), 304 coarse grain stainless steel (CG-SS), and 304 nano-grain austenitic stainless steel (NG-SS) that the relationship between Vickers hardness and nanoindentation hardness does not conform to mathematical geometric relationships, owing to the sink-in and pile-up effects. This intricate non-linear relationship poses significant challenges in predicting Vickers hardness.

Discerning latent relationships among parameters is an area where neural networks excel remarkably. Neural networks have experienced substantial advances within the domain of materials science [32,33,34,35,36,37,38,39,40,41,42,43], with notable breakthroughs in facets such as materials genomics and predictive modeling. Such networks have been harnessed to predict properties of a diverse array of materials, encompassing but not confined to varied compositions of steel, hence propelling the progression of material design and discovery [44,45,46,47,48]. Furthermore, neural networks have been employed to predict mechanical performance and behavior under disparate conditions, offering invaluable insights that aid the design and manufacturing processes. Karimi et al. [49] developed a Graph Neural Network (GNN) model focused on predicting the nanoscale hardness of 310S steel. Utilizing grain location and orientation data, the model learns from nanomechanical load-displacement curves, effectively predicting the material’s micromechanical response. The findings not only provide a rapid and cost-effective method for hardness estimation but also guide more detailed nanoindentation experiments. Moreover, this research underscored the complementary nature of data-driven methods to traditional laboratory measurements and physical simulations, marking a significant contribution to the field of materials science. In this context, our predictive model, duly trained on a substantial dataset of corresponding Vickers hardness and nanoindentation hardness measurements, is projected to contribute significantly to this continuous trend.

In this research, we introduce an innovative approach that employs deep neural network technology to predict the Vickers hardness (HV) values of materials from nanoindentation experiments. Our goal was to construct and train a robust neural network model based on detailed HV and nanoindentation hardness measurement data. Building upon this, we innovatively generated a dataset spanning a hardness gradient of 400 to 1000 HV by subjecting M50NiL steel to a dual process of carburization and nitriding. This model focuses on the prediction of Vickers hardness within this hardness gradient range, with an emphasis on assessing its interpolation accuracy within this interval. To determine the optimal prediction model, we compared four contemporary neural network architectures: Multilayer Perceptron (MLP), Convolutional Neural Network (CNN), Long Short-Term Memory network (LSTM), and Transformer. After a series of tests and evaluations, Multilayer Perceptron (MLP) was selected as the best model due to its exceptional performance in predicting Vickers hardness. The advancements of this study not only enhance our characterization and understanding of the properties of thin films and coating materials but also provide significant technological innovation in the measurement of hardness within the field of materials science.

## 2. Methodology

### 2.1. Collection of Data

In the quest for securing a substantial number of hardness data points, the fabrication of an ample assortment of hardness standard specimens becomes a prerequisite. Nonetheless, the voluminous data requirement of neural networks presents an obstacle, rendering this approach practically unattainable. In the pursuit of an adequate hardness dataset, we designed an innovative strategy: through the dual treatment of carburizing and nitriding applied to M50NiL steel [50,51,52], we successfully procured a subtle hardness gradient extending from the surface to the core. Our choice of M50NiL steel was driven by its widespread use as a bearing material, reflecting its industrial relevance. Furthermore, the ability of M50NiL steel to achieve a wide range of hardness levels through these treatments allowed us to generate a broad and diverse dataset, crucial for the accurate training of our neural network models. This strategic choice not only enhanced the breadth and applicability of our dataset but also ensured robust and wide-ranging applicability of our models in real-world scenarios, particularly in industries where M50NiL steel is prevalently used.

#### 2.1.1. Materials and Surface Treatment

The material under investigation was M50NiL steel, the constituents of which are elucidated in Table 1. The specimens employed were annealed cylindrical entities, exhibiting dimensions of Φ35 × 10 mm. The initial carburizing procedure was executed at 960 °C for a period of 20 h, followed by a 2-bar gas quenching process. Subsequent to this was a high-temperature quenching protocol, with a specified quenching temperature of 1100 °C, an isothermal hold duration of 40 min, and a quenching pressure of 2 bar. The carburizing process leverages a vacuum carburizing furnace (ECM, ICBP^®^ Flex, Grenoble, France), while the quenching phase employs a miniature high-temperature vacuum furnace (BMI, BMICRO, FL, USA). Plasma nitriding treatment ensued, conducted within a glow ion nitriding furnace, an item of equipment custom-built by our research group. Plasma nitriding was facilitated through the active screen nitriding technique, thereby achieving a remarkably uniform nitriding layer [53,54,55]. A pulsed power supply (bias of 800 V, 40 kHz, adjustable duty cycle) was utilized for nitriding treatment. In the absence of power supply activation and gas infusion, the chamber was evacuated to a pressure of 5 Pa. The determined experimental parameters included an N_2_:H_2_ ratio of 1:20, a temperature of 500 °C, and a duration of 50 h. Upon the conclusion of nitriding, both the nitriding furnace and the samples underwent a gradual cooling process to room temperature.

#### 2.1.2. Hardness Test

**Figure 1:**
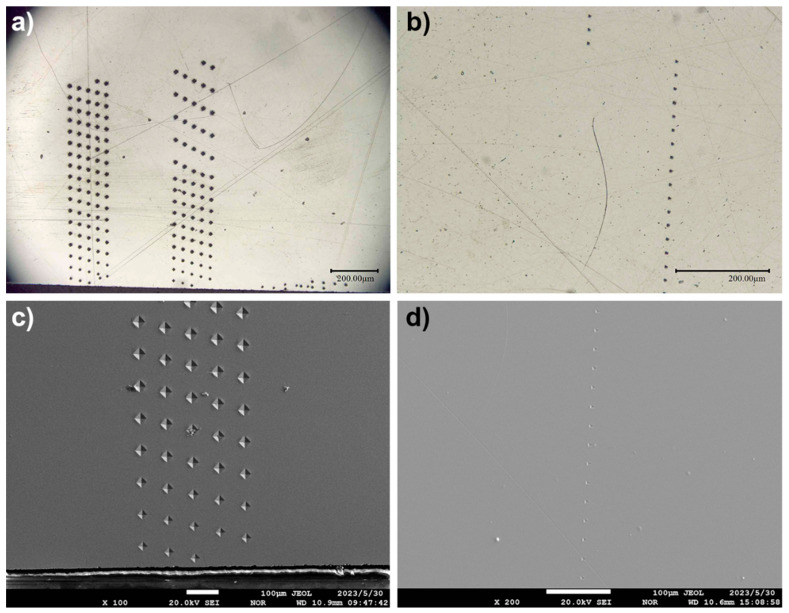
OM and SEM images used for calibrating indenter positions in Vickers and nanoindentation hardness tests. (**a**,**b**) are OM images for Vickers and nanoindentation tests; (**c**,**d**) are SEM images for the same.

Prior to the execution of hardness testing, all specimens underwent ultrasonic cleansing within an ethanol medium. Subsequent to this, surface cross-sectional hardness examinations were conducted. The post-nitriding micro-Vickers hardness gradient was quantified utilizing a Micro Vickers Hardness Tester (Buehler, VH3100, Bluff, IL, USA), operating under a load of 500 g for a duration of 15 s. This process facilitated the acquisition of between 300 and 400 Vickers hardness data points at specified depths along the cross-section. All data points used were subjected to error analysis, and data points with varying errors were incorporated into the datasets. Nanoindentation hardness was assessed via the Continuous Stiffness Measurement (CSM) method, enabling the detection and subsequent elimination of anomalous data points throughout the testing process. This resulted in a final tally of 90 nanoindentation data points at predetermined depths. As illustrated in Figure 1, by utilizing Optical Microscopy (OM) and Scanning Electron Microscopy (SEM), we calibrated the locations for two types of hardness test points, thereby obtaining an accurate relationship between position and hardness. Specifically, Figure 1a,b represent the OM images of Vickers hardness and nanoindentation hardness, respectively, while Figure 1c,d are partial images of the SEM for Vickers hardness and nanoindentation hardness, respectively. These two datasets served as the foundation for the generation of subsequent datasets.

### 2.2. DNN Model

**Figure 2:**
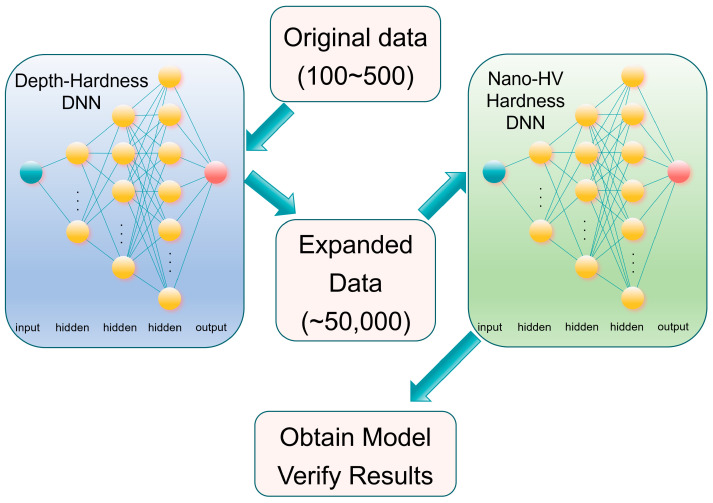
Schematic representation of the DNN model-development process.

The comprehensive model workflow is depicted in Figure 2. Through the implementation of the two data sets, separate DNN models correlating depth with both Vickers hardness and nanoindentation hardness were established. Utilizing these two models allowed for the procurement of extended datasets corresponding to both Vickers and nanoindentation hardness. Ultimately, a predictive model was achieved, facilitating the determination of Vickers hardness based on nanoindentation hardness. In this study, to ensure uniformity in experimental conditions, all deep learning models were trained on graphics cards (NVIDIA, GeForce RTX 4080, Santa Clara, CA, USA), which possess equal computational capabilities. Such a practice helped to eliminate the potential impact of hardware differences on the experimental outcomes. Furthermore, the use of TensorFlow, a widely recognized open-source framework, for building and training the models ensured the standardization and reproducibility of the entire research process.

#### 2.2.1. Depth-Hardness Model

In the current study, we applied deep learning techniques to accurately construct predictive models for nanoindentation and Vickers hardness. The model, implemented using the TensorFlow library’s neural network architectures [56,57,58], was specifically optimized for the complex relationship between hardness characteristics and material depth. In an environment equipped with high-performance Graphics Processing Units (GPUs), the model underwent a rigorous training process utilizing a specially collected dataset of hardness tests. Our network utilized the depth of the material layers as input, with the prediction target of corresponding hardness values.

Prior to the formal training, we conducted thorough preprocessing of the dataset, which included data cleansing, outlier handling, and the partitioning into training, validation, and test sets. To negate the impact that may arise from different scales of measurement, we further implemented normalization of the input features. During this process, the *StandardScaler()* method from the scikit-learn library was employed.

The neural network model used in our study consisted of several fully connected layers (dense layers), including an input layer, three hidden layers, and an output layer. The arrangement of neurons in our hidden layers, set at 32, 64, and 128 units, respectively, followed a well-established principle in neural network architecture. This progression allowed our model to incrementally capture more complex features, with each layer doubling its neuron count to effectively handle the increasing complexity of data representation. This design ensured a harmonious balance between computational efficiency and the ability to intricately model the characteristics of our dataset. The sigmoid function was chosen as the activation function to effectively address non-linear problems. For model training, the Adam optimizer was utilized, with the mean squared error (MSE) loss function serving as the optimization target. The mean absolute error (MAE) was also monitored to assess the quality of training.

During the training phase, the model underwent 5000 iterations of learning, with a large batch size of 1024 implemented to execute the batch gradient descent algorithm. Additionally, custom callback functions were utilized to track training progress and model performance. Following rigorous testing and validation, this depth-hardness model was successfully saved and confirmed to have stable and superior performance characteristics.

#### 2.2.2. Nanoindentation-Vickers Hardness Dataset

To overcome the limitation of insufficient original hardness data, our study adopted an innovative strategy, namely, the augmentation of the dataset using deep learning techniques through an established depth-hardness prediction model. The two TensorFlow-based neural network models, once trained, were capable of predicting hardness values at varying depths. Using these models, we generated 25,000 data points covering a depth range from 100 to 1800 nanometers. These data points comprehensively reflected the quantitative relationship between nanoindentation hardness and Vickers hardness, and their integration into an expanded dataset not only enriched the training material but also extended the model’s applicability beyond the specific steel initially used. This approach significantly enhanced the model’s generalizability and predictive accuracy for a wider range of materials. The generated data points were integrated to form an expanded dataset, intended to provide the neural network with ample training resources to ensure the model’s generalizability and predictive accuracy.

In this section, further explanation is required regarding the generation of these data points and how they contribute to enhancing the model’s generalization capability and predictive accuracy. Initially, these data points were generated through nanoindentation experiments on M50NiL steel subjected to carburizing and nitriding, as this process yielded a wide range of hardness data points (400–1000 HV). This ensured that our generated data covered a broad spectrum of practical application scenarios. Regarding the construction of the model, although it utilized only a few hundred data points, these points were not entirely random. They exhibited a certain continuity or conformed to a specific curve relationship. This characteristic enabled us to generate a large number of hardness values at various depths using the model’s derived curves from a limited amount of data. This implied that the model learned not only the hardness values but also how these values vary with changes in the material’s microstructure and testing conditions. Finally, by integrating these simulated data points with the original dataset, we significantly increased the volume and diversity of data available for model training. This aspect was crucial for enhancing the model’s generalization capability. Generalization capability refers to the model’s ability to adapt and predict new data that it has not previously encountered. In our case, this meant that the model could accurately predict the hardness of various materials, even if those materials were not included in the original dataset.

#### 2.2.3. Nanoindentation–Vickers Hardness Model

Building on the dataset established in Section 2.2.2, our study meticulously evaluated the performance of four different structured neural network models in the prediction of hardness. These models included MLP, CNN, LSTM, and Transformer. Despite differences in their architectural structures, all models were constructed and optimized following the fundamental design principles of the depth-hardness model outlined in Section 2.2.1.

During the model evaluation process, key metrics such as training time consumption, response speed, mean squared error (MSE), and the coefficient of determination (R^2^) were given special attention, while the models’ accuracy on the test set was also examined. By synthesizing these performance indicators, this study identified the most suitable neural network model for predicting the relationship between nanoindentation and Vickers hardness through comparative analysis. The selected model possessed excellent predictive capabilities and computational efficiency, meeting the demands for precise and rapid hardness prediction.

## 3. Results and Discussion

**Figure 3:**
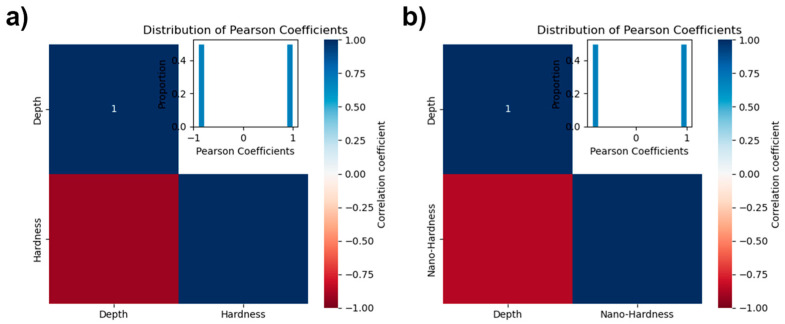
Pearson correlation charts for (**a**) nanohardness and (**b**) Vickers hardness against nitriding depth. Correlation coefficients are −0.863 for nanohardness and −0.911 for Vickers hardness, indicating a strong negative correlation with depth for both hardness measures.

The distance-hardness datasets for nanoindentation and Vickers hardness were obtained using a method of cross-validation between OM and SEM images. Figure 3a,b display the Pearson correlation graphs for nanoindentation and Vickers hardness relative to nitriding depth, with correlation coefficients of −0.863 and −0.911, respectively. This high degree of correlation for both types of hardness with respect to depth indicates that establishing neural network models was highly appropriate for this context.

**Figure 4:**
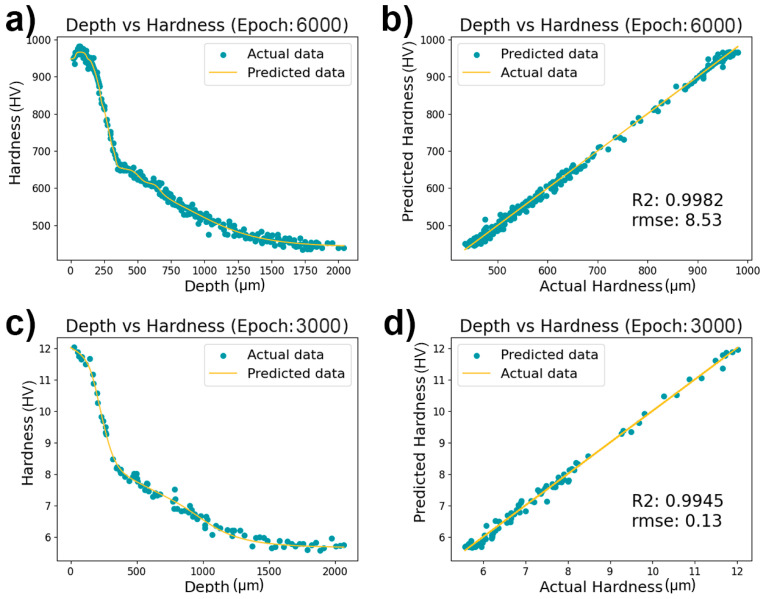
Neural network model predictions for depth vs. hardness. (**a**) Depth-hardness curve for Vickers hardness, (**b**) actual vs. predicted Vickers hardness, (**c**) depth-hardness curve for nanoindentation hardness, (**d**) actual vs. predicted nanoindentation hardness. Models were trained with early stopping to avoid overfitting, showing high correlation between predictions and actual measurements.

By establishing DNN models, we generated depth-hardness curves and compared actual versus predicted hardness profiles. To address the limited data available for our neural network model, and to preserve clarity in our graphical representations, all data points—including those from the training, validation, and test sets—were plotted collectively. This approach allowed us to provide a clear, comprehensive view of the model’s performance across the entire dataset. Figure 4 illustrates the relationship between depth and hardness, as predicted by our neural network models, with Figure 4a,b representing the depth-hardness and actual-predicted curves for Vickers hardness, respectively. Figure 4c,d correspond to the depth-hardness and actual-predicted curves for nanoindentation hardness, respectively. Both models were trained through 3000 and 6000 iterations, utilizing early stopping mechanisms to prevent overfitting. It is evident from Figure 4 that the prediction data increasingly converged with the actual data as the number of iterations rose. After training with 3000 iterations, the nanoindentation hardness model achieved an R^2^ value of 0.9945, indicating an exceptionally high correlation between the model predictions and the actual values. The corresponding RMSE was 0.13, signifying a relatively small discrepancy between predicted and actual values. For the Vickers hardness model, an R^2^ value of 0.9982 was attained after 6000 iterations of training, suggesting that the model could explain nearly all the variability. The RMSE for this model stood at 8.53, which was also comparatively low. Overall, the performance of both models was quite impressive, underscoring the effectiveness of employing neural networks for hardness modeling. This approach can be harnessed for the prediction of hardness and the analysis of material properties.

### 3.1. Validation of Nanoindentation–Vickers Hardness DNN Model

**Figure 5:**
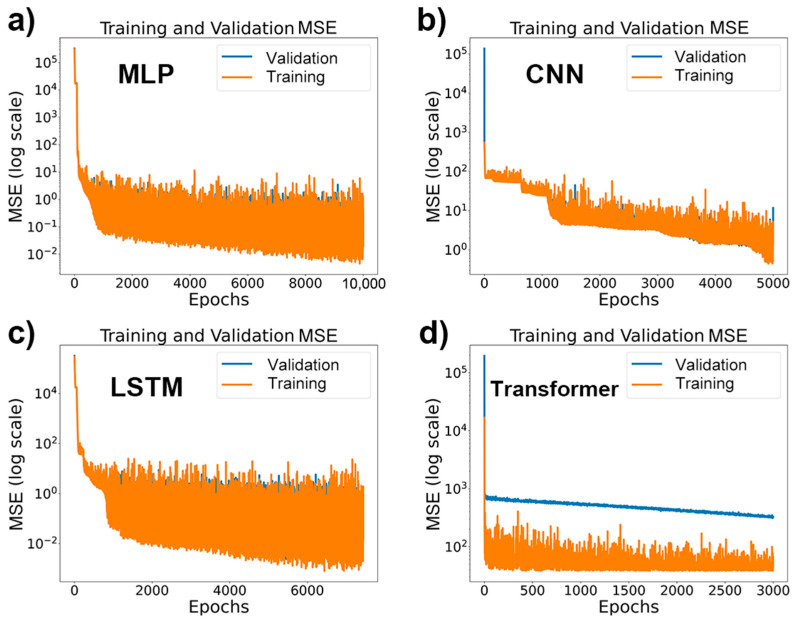
Training and validation loss curves for neural network models on a nanoindentation–Vickers hardness dataset. (**a**) MLP model showing loss over 10,000 epochs; (**b**) CNN model with loss over 5000 epochs; (**c**) LSTM model depicting loss across 7500 epochs; (**d**) Transformer model’s loss observed for 3000 epochs. Each subplot illustrates the initial rapid decrease in loss, followed by stabilization or fluctuations as training progresses.

Utilizing two depth-hardness models, we generated a dataset comprising 25,000 pairs of nanoindentation and Vickers hardness data. A neural network model was established to analyze this dataset. Validation was conducted on four distinct neural network architectures—MLP, CNN, LSTM, and Transformer—from which we first obtained their training curves, as depicted in Figure 5. The experiment monitored the models’ loss curves throughout the training process, including both training and validation losses. In all cases, the training curves exhibited similar trends. Initially, both training and validation losses decreased rapidly, indicating that the models were effectively learning patterns from the training data during the initial learning phase. As the number of training epochs increased, the training loss continued to decrease, whereas the validation loss exhibited some fluctuations. These oscillations can arise when a model, particularly if it is of a high capacity, learns inconsistent features across different data subsets, introducing volatility into the loss curves. Despite these fluctuations, the overall trend was a decrease, signifying that the models were improving their performance on the validation set throughout the training process. More importantly, these oscillations did not necessarily affect the final experimental outcomes. Experimental results typically depend on a model’s final performance, rather than on every step of the entire training process. As long as there was a general downward trend in the MSE on the validation set, the model was considered to be improving.

**Figure 6:**
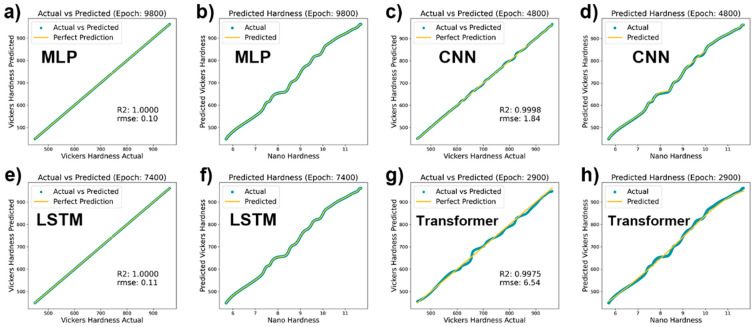
Comparison of machine learning models for Vickers hardness prediction: (**a**) MLP accuracy, (**b**) MLP predicted vs. nanohardness, (**c**) CNN accuracy, (**d**) CNN predicted vs. nanohardness, (**e**) LSTM accuracy, (**f**) LSTM predicted vs. nanohardness, (**g**) Transformer accuracy, (**h**) Transformer predicted vs. nanohardness.

Upon evaluation of the four fully trained models, as illustrated in Figure 6, the MLP, CNN, LSTM, and Transformer demonstrated varying performance in predicting Vickers hardness. The MLP model showed near-perfect prediction in both the actual vs. predicted plots (Figure 6a) and the predicted hardness plot (Figure 6b), achieving an R^2^ value of 1.0000 and an RMSE of just 0.10, indicating extremely high accuracy and minimal error in its predictions. The CNN model, with an R^2^ value of 0.9998 and an RMSE of 1.84, as shown in the actual vs. predicted plots (Figure 6c), also displayed high prediction precision in the predicted hardness plot (Figure 6d), although it was slightly less precise compared to the MLP model. The LSTM model maintained an R^2^ value of 1.0000 and an RMSE of 0.11 in the actual vs. predicted plots (Figure 6e), showcasing prediction capabilities on par with those of the MLP, and exhibited similar prediction trends to those of the MLP and CNN in the predicted hardness plot (Figure 6f). Lastly, the Transformer model’s performance, with an R^2^ value of 0.9975 and an RMSE of 6.54 in the actual vs. predicted plots (Figure 6g), although consistent with the actual hardness trend in the predicted hardness plot (Figure 6h), indicated a larger prediction error compared to those of the other models.

In summary, the MLP and LSTM models exhibited the highest accuracy and lowest error in predicting Vickers hardness, while the Transformer model performed slightly less well on these metrics. Interestingly, the LSTM model demonstrated high accuracy and low error in the predictions, which may be attributed to its design for handling and predicting significant events in time-series data, as it can learn long-term dependencies. This suggests that the Vickers hardness data may contain time-series characteristics or sequential patterns, which the LSTM is capable of capturing, thereby enhancing its predictive performance.

**Figure 7:**
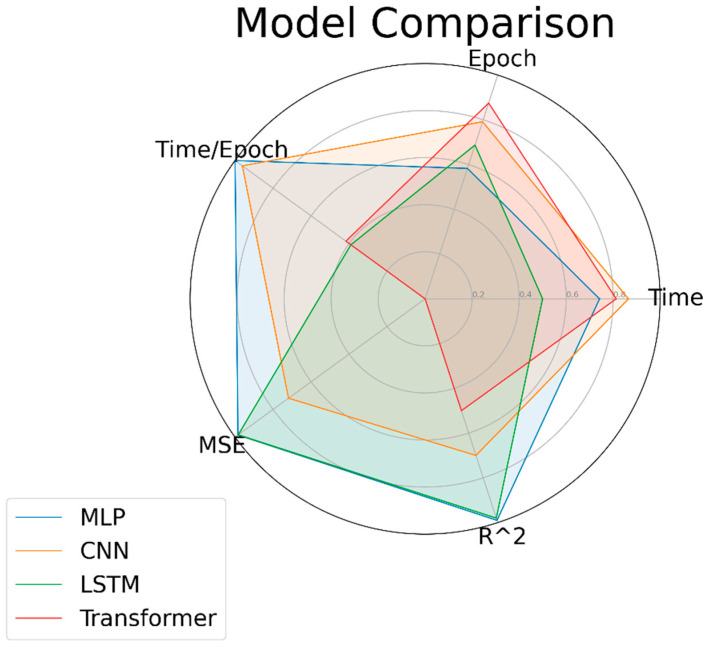
Radar chart comparing the performance of MLP, CNN, LSTM, and Transformer models on metrics of time, epochs, time per epoch, MSE, and R^2^.

Table 2 and the corresponding radar chart (Figure 7) present a comparative performance analysis of the four neural network models: MLP, CNN, LSTM, and Transformer. The MLP had the shortest duration per iteration (0.25 s) and boasted the lowest mean squared error (MSE = 0.10) coupled with a perfect coefficient of determination (R^2^ = 1.00), indicating its efficiency and accuracy. Although the total training time for the MLP was relatively long (2469.2 s) and required a high number of iterations (10,000), it demonstrated high efficiency per iteration. In contrast, the CNN had a shorter total training time but less accuracy (MSE = 1.84). The LSTM was comparable to the MLP in terms of accuracy but had the longest training duration (4793.6 s). The Transformer, while faster, lacked in accuracy (MSE = 6.54), leading to its minimal representation on the radar chart in Figure 7. This visual anomaly was due to the scale of MSE values, where the Transformer’s significantly higher MSE resulted in a much lower score on the radar chart. Upon comprehensive consideration, given its outstanding iterative efficiency and accuracy, the MLP was the most suitable model under the conditions of this study.

Previous studies posited that the relationship between nanoindentation hardness and Vickers hardness is generally linear [59,60,61]. Observations from Figure 6 also suggest an approximately linear relationship. This correlation may persist regardless of its linearity due to material characteristics, testing errors, and inherent coupling effects. The capacity to refine accuracy by incorporating more data is precisely where the strength of neural networks lies. This adaptability to enhance precision with additional data input demonstrates the neural networks’ robustness in modeling complex relationships, even when they deviate from linearity.

### 3.2. Experimental Verification of DNN Models

The model developed in this study was validated on an established dataset to ensure its predictive accuracy within a specific parameter range. To further investigate the robustness of our model in predicting the hardness of M50NiL steel materials, our team implemented a series of laboratory-level validation measures. Moreover, to comprehensively evaluate the model’s generalization capabilities, we compared the performance of different steel grades under identical testing conditions. Table 3 and Table 4 document the data for these materials from Vickers hardness tests. Due to space constraints, not all test data could be listed in the tables. Within the hardness range designed for the model, namely 400–1000 HV, the model demonstrated a relatively precise predictive capability, with an error rate below 4%. However, beyond the dataset’s hardness range, particularly in regions above 1000 HV, the model’s prediction accuracy significantly decreased, revealing limitations when extrapolating to uncovered data. We believe that by further expanding the dataset to encompass a broader range of hardness, this issue might be effectively addressed.

## 4. Conclusions

In summary, our research on the prediction of hardness in M50NiL steel using deep neural network models led to several key findings:

- Model Performance Comparison: The MLP and LSTM models demonstrated superior performance in terms of accuracy and error metrics, with the MLP showing particularly commendable iteration efficiency and precision in prediction. While the CNN model benefited from shorter training times, its accuracy was comparatively lower. The Transformer model was notably deficient in accuracy.
- Applicability Across Steel Varieties: The developed predictive models proved effective not only for M50NiL steel but also for other types of steel, indicating a broad adaptability. This suggests that the neural network models we developed hold the potential for widespread application in the field of materials science.
- Advancement in Measurement Techniques: This study supports the adoption of nanoindentation as a direct measurement method for HV hardness, particularly apt for thin films and areas with significant gradient variation. This method offers a more precise and convenient approach to determining hardness in materials with complex microstructures.

The demonstrated efficacy of MLP and LSTM models for predicting the hardness of various steels paves the way for the development of more sophisticated, real-time predictive algorithms in materials science. The potential expansion of these models to a wider range of materials, coupled with the precision offered by nanoindentation techniques, heralds a future of enhanced material design and smarter manufacturing processes tailored to specific performance criteria.

All the DNN models and codes mentioned in this study have been uploaded to the Supplementary Material section for the convenience of readers who wish to delve deeper into and utilize these technologies.

## Figures and Tables

**Table 1:** Chemical composition (in wt.%) of M50NiL steel.

C	Cr	Mo	V	Ni	Mn	Si	Fe
0.13	4.1	4.2	1.2	4.2	0.13	0.18	Bal.

**Table 2:** Performance comparison of four different machine learning models—MLP, CNN, LSTM, and Transformer—in terms of training time, number of epochs, time per epoch, coefficient of determination (R^2^), and mean squared error (MSE) for predicting Vickers hardness within the range of 400–1000 HV.

	Time (s)	Epoch	Time/Epoch(s)	MSE	R^2^
MLP	2469.2	10,000	0.25	0.10	1.0000
CNN	1291.8	5000	0.26	1.84	0.9998
LSTM	4793.6	7500	0.64	0.11	1.0000
Transformer	1790.5	3000	0.60	6.54	0.9975

**Table 3:** Predicted, actual, and error values for the Vickers hardness within the range of 400–1000 HV.

	M50NiL	M50NiL Carburizing				M50NiL Carburized and Nitriding			M50	M50 Nitriding		Others
Predicted	453.6	517.4	651.8	660.2	713.0	819.3	924.5	965.6	750.5	842.8	924.8	/
Actual	460	515	645	670	730	830	940	1000	740	860	935	/
Errors	1.40%	0.46%	1.05%	1.47%	2.33%	1.33%	1.70%	3.50%	1.41%	2.01%	1.10%	1.80%

**Table 4:** Predicted, actual. and error values for Vickers hardness outside the range of 400–1000 HV.

	M50 Nitriding Compound Layer			M50 Nitriding Diffusion Layer			Others		
Predicted	965	965	965	965	965	965	/	/	/
Actual	1350	1340	1290	1100	1110	1090	/	/	/
Errors	28.52%	27.99%	25.19%	12.27%	13.06%	11.47%	30.29%	27.38%	41.56%

## Data Availability

Data are contained within the article and Supplementary Material.

## Supplementary Materials

The following supporting information can be downloaded at: https://www.mdpi.com/article/10.3390/ma17010148/s1.
